# Incidence of pulmonary tuberculosis in Chinese adults with type 2 diabetes: a retrospective cohort study in Shanghai

**DOI:** 10.1038/s41598-020-65603-y

**Published:** 2020-05-22

**Authors:** Yanyun Li, Juntao Guo, Tian Xia, Fei Wu, Jingyan Tian, Minna Cheng, Wanghong Xu, Qinping Yang, Jing Chen, Zheyuan Wu, Qinghua Yan, Yan Shi, Fan Wu

**Affiliations:** 1 Division of Chronic Non-communicable Disease and Injury, Shanghai Municipal Center for Disease Control and Prevention, Shanghai, 200336 China; 2 Division of Tuberculosis Control, Shanghai Municipal Center for Disease Control and Prevention, Shanghai, 200336 China; 3 Division of Public Health Informatics, Shanghai Municipal Center for Disease Control and Prevention, Shanghai, 200336 China; 4 Department of Endocrinology and Metabolism, Shanghai Institute of Endocrine and Metabolic Diseases, Ruijin Hospital, Shanghai Jiao Tong University School of Medicine, Shanghai, 200025 China; 5 School of Public Health, Shanghai Medical College, Fudan University, Shanghai, 200032 China; 6 National Clinical Research Center for Aging and Medicine, Huashan Hospital, Fudan University, Shanghai, 200040 China; 7 Shanghai Medical College, Fudan University, Shanghai, 200032 China

**Keywords:** Type 2 diabetes, Tuberculosis

## Abstract

To estimate the incidence of pulmonary tuberculosis (PTB) in Chinese diabetes patients and to evaluate the effect of blood glucose on PTB risk, a retrospective cohort study was built based on the diabetes management system in Shanghai and included 240,692 adults aged 35 or above. Incidences of PTB in all diabetes patients and by subgroups were calculated and compared. Multivariable Cox regression models with restricted cubic splines were used to evaluate the association of fasting plasma glucose (FPG) with the risk of PTB. A total of 439 incident PTB cases were identified in the cohort after an average of 3.83 years of follow-up. The overall PTB incidence rate was 51.3/100,000 in diabetes patients, and annual incidence remained higher than that in general population. The PTB incidence rate of diabetes patients was higher in men than in women (86.2 vs. 22.1 per 100,000) and was highest in patients with body mass index (BMI) < 18.5 kg/m^2^ (215.2/100,000) or FPG ≥ 10 mmol/L (143.2/100,000). Our results suggest that the risk of tuberculosis may be greater at higher levels of FPG in diabetes patients of normal weight. Specific tuberculosis screening strategies for different characteristic diabetes population should be provided to prevent and control tuberculosis in China.

## Introduction

Tuberculosis (TB) remains a major global public health issue despite its slowly decreased incidence, particularly in developing countries. The World Health Organization (WHO) reported that an estimated 10 million people developed TB and 1.6 million people died of TB worldwide in 2017^1^. At the same time, type 2 diabetes mellitus (T2DM) is a global epidemic. It is estimated that there were around 425 million prevalent diabetes patients worldwide in 2017, and the number is expected to be almost 700 million by 2045^2^.

The associations of T2DM with the incidence, severity and clinical outcome of TB have been well documented^3–7^. The estimated number of incident TB attributable to T2DM increased from 10% in 2010 to 15% in 2013, globally, and almost 17% of diabetes-associated TB cases occurred in China^8^. A recent meta-analysis demonstrated that T2DM was associated with a two- to four-fold increased risk of active TB^9^. Shanghai, a typical megacity of China, experiencing rapid population aging, urbanization, industrialization and changes in lifestyle, is bearing heavy dual burden of prevention and control for diabetes and TB. Although TB incidence rate is relatively low in Shanghai, approximately 26.9 per 100,000 in 2015, the prevalence of diabetes is dramatically increasing. A cross-sectional investigation in 2013 reported that overall weighted prevalence of diabetes was 17.6% among Shanghai residents aged 35 or above, which was much higher than the nationwide average level^10^.

Therefore, to realize the WHO’s END TB Strategy, which was approved by the 67^th^ World Health Assembly in 2014^11^, the Shanghai Municipal Commission of Health and Family Planning launched “Plan for Hierarchical Diagnosis and Treatment and Comprehensive Prevention and Treatment of Tuberculosis” in 2017, particularly addressing the implementation of screening for TB in patients with T2DM. However, the validity and reliability of screening for TB among T2DM were not convincing due to the lack of well-determined screening criteria and methods. A prospective study assessing the screening for TB in T2DM patients in community health settings in Kunming, China, found only a small number of TB cases were diagnosed among the T2DM patients with a positive symptom screen^12^. Owing to limited medical resources and relatively low incidence rate in Shanghai, it is important to identify risk factors of TB in T2DM patients in order to obtain more beneficial effectiveness.

Additionally, several studies have suggested that adequate management of blood glucose would have a significant positive influence on the reduction of TB incidence and mortality^13–15^. However, on the one hand, published studies on comprehensive epidemics of TB among T2DM patients in China are considerably insufficient. On the other hand, evidence on the association between TB incidence risk and blood glucose is still not consistent worldwide^16–18^.

Given the above situation, our study was aimed to analyse epidemiological characteristics of pulmonary tuberculosis (PTB) in T2DM patients and its association with fasting plasma glucose (FPG), and eventually to optimize TB screening strategies among T2DM patients in Shanghai, China.

## Methods

### Data sources

The diabetes patient data used in the study were from Shanghai Standardized Diabetes Management System (SSDMS) operated by Shanghai Municipal Center for Disease Control and Prevention. The system was initiated in 2004 and well-established after the launch of the National Basic Public Health Service Program (NBPHSP) in 2009^19^. According to the requirement of the NBPHSP, in Shanghai, community health centres (CHCs) take responsibility to provide management for T2DM patients aged 35 years or above and to upload the electronic records to SSDMS. Nearly half of all diagnosed T2DM cases in Shanghai were registered in the system^10^, including newly diagnosed cases through community-based screenings or physical examinations and previously diagnosed cases through routine outpatient visits. Baseline information for each T2DM case in SSDMS, including height, weight, waist and hip circumference, blood pressure and blood glucose, were collected through an initial assessment before being registered.

Incident PTB of managed T2DM patients was identified through the record linkage to the Shanghai TB surveillance system and mandatory reporting system using the unique identification card number for each individual. The Shanghai TB surveillance and mandatory reporting system has been established since the 1990s, covering all PTB patients in Shanghai. Each TB patient in the system was confirmed by physicians in designated TB hospitals/clinics through conducting a sputum smear test and bacterial culture.

The deaths of managed T2DM patients were identified by annual record linkage to the Shanghai Vital Statistics Registry during the whole study period.

This study was approved by the Ethical Review Committee of Shanghai Municipal Center for Disease Control and Prevention. Since this was a retrospective study and all patients’ information used was routinely collected through the SSDMS, the Shanghai TB surveillance and mandatory reporting system and the Shanghai Vital Statistics Registry, requirement for informed consent was exempted by the committee mentioned above.

### Study design and population

A retrospective cohort was established based on the Shanghai Standardized Diabetes Management System. By the end of 2015, a total of 552,377 T2DM patients had been registered in the system. In this study, we excluded 311,685 patients diagnosed with T2DM before January 1, 2010. Ultimately, a total of 240,692 T2DM patients were included in the present analyses. The patients were censored as lost to follow-up if they died or moved out of Shanghai.

### Related definitions

T2DM patients were defined as (1) FPG level of 7.0 mmol/L or higher, or (2) 2-h plasma glucose (venous plasma glucose 2 h after ingestion of 75 g oral glucose load) level of 11.1 mmol/L or higher according to the 1999 WHO criteria^20^. The subgroups of FPG level were defined for certain analysis: <4.4 mmol/L, 4.4–5.9 mmol/L, 6.0–7.9 mmol/L, 8.0–9.9 mmol/L and ≥10.0 mmol/L according to the Chinese expert consensus on management of diabetes in Chinese adults^21^.

PTB cases were defined as individuals who had positive sputum smear, positive sputum culture, or pulmonary lesions of tuberculosis that had been confirmed by pathological examination according to the Guideline for National TB Control Program^22^. Additionally, in our study, only individuals whose date of diagnosis with PTB was posterior to the date of diagnosis with T2DM could be confirmed as PTB cases.

Body mass index (BMI) was calculated as weight (kg) divided by the square of height (m). The diabetes patients were classified into three groups according to BMI level: underweight (<18.5 kg/m^2^), normal weight (18.5–24.9 kg/m^2^) or overweight/obese (≥25 kg/m^2^)^23^. Central obesity was defined as waist circumference over 90 cm for men or more than 85 cm for women. High blood pressure was defined as systolic blood pressure ≥140 mmHg or diastolic blood pressure ≥90 mmHg.

### Statistical analysis

The annual incidence of PTB among T2DM patients in the cohort from 2010 to 2015 was calculated as the number of PTB cases divided by the average number of patients, which was estimated based upon the population numbers at the start and end of each year. The age-standardized rates per 100,000 for each year were calculated using the direct method, based on the 2010 Shanghai standardized population by 5-year age groups from the Shanghai Vital Statistics Registry and age-specific annual incidence. The annual incidence in the general population from 2010 to 2015 was calculated by the number of incident PTB divided by the number of adults aged 35 or above in the Shanghai population in a given year. Person-years (PYs) of follow-up were calculated from the date that T2DM was first diagnosed to the date of diagnosis with PTB, the date of loss to follow-up, or the end of the study, December 31, 2015, whichever occurred first. The overall or group-specific incidence rate of PTB was calculated by the number of incident PTB cases divided by person-years of follow-up.

Comparison of annual incidence of PTB between T2DM patients and the general population was conducted using a *Z* test. A log-rank test was used to examine the difference of PTB incidence rate across subgroups, and a Kaplan-Meier survival curve was used by FPG category. The Cox proportional hazards regression model was applied to access the association between FPG and risk of PTB incidence. FPG was used as a continuous variable as well as a categorical variable and confounding variables including age, sex and BMI were adjusted in the model. The potential non-linear relationship was also detected among all T2DM patients and by sex using restricted cubic splines (RCS) with the 5th, 50th and 95th percentiles as fixed knots^24^ and FPG was used as a continuous variable. Two statistical tests were performed through the RCS procedure. The first null hypothesis was that the regression coefficients of both linear and non-linear terms of the factor were equal to zero, and the result was presented as “*P* for overall association”. The other one was the test of the regression coefficient of nonlinear term, and the result of “*P* for non-linearity” <0.05 implied a non-linear association.

Statistical analyses were performed using SAS version 9.4 (SAS Institute, Inc., Cary, North Carolina). RCS was completed by SAS macro%RCS. P < 0.05 was considered statistically significant.

## Results

As shown in Table 1, a total of 110,262 male (45.8%) and 130,430 female (54.2%) T2DM patients were included in this study. The proportion of T2DM patients aged 65 years or older increased continuously from 36.4% in 2010 to 49.9% in 2015. The proportion of women remained higher than men over the six calendar years.

**Table 1:** Description of newly enrolled and eligible T2DM patients in the cohort by calendar year.

N (%)	2010	2011	2012	2013	2014	2015	P value*
Overall	48 289 (20.1)	47 506 (19.7)	49 125 (20.4)	39 133 (16.3)	32 836 (13.6)	23 803 (9.9)	
Age group (years)							<0.001
35–44	1801 (3.7)	1650 (3.5)	1606 (3.3)	1197 (3.1)	915 (2.8)	610 (2.6)	
45–54	10 699 (22.2)	9427 (19.8)	8846 (18.0)	5862 (15.0)	4543 (13.8)	2873 (12.1)	
55–64	18 197 (37.7)	17 920 (37.7)	18 263 (37.2)	14 857 (38.0)	12 245 (37.3)	8452 (35.5)	
65–74	10 953 (22.7)	11 073 (23.3)	12 347 (25.1)	10 152 (25.9)	8881 (27.1)	6903 (29.0)	
75–	6639 (13.8)	7436 (15.7)	8063 (16.4)	7065 (18.1)	6252 (19.0)	4965 (20.9)	
Sex							0.001
Men	21 843 (45.2)	21 557 (45.4)	22 801 (46.4)	17 929 (45.8)	15 110 (46.0)	11 022 (46.3)	
Women	26 446 (54.8)	25 949 (54.6)	26 324 (53.6)	21 204 (54.2)	17 726 (54.0)	12 781 (53.7)	

*P values were calculated using a Chi-square test.

A total of 439 incident PTB cases were identified during the study period. As presented in Table 2, the annual incidence of PTB in T2DM patients did not change substantially from 2010 to 2015 and was significantly higher than in the general population except in 2010. While the annual incidence and age-standardized rate (ASR) of PTB in T2DM patients ranged from 76.4 and 99.9/100,000 in 2011 to 44.5 and 50.8/100,000 in 2015, respectively, the incidence in the general population was around 30.0/100,000 during the six years. The incidence was much higher in men than in women in both T2DM patients and general population.

**Table 2:** Comparison of annual incidence of PTB/100000 population in T2DM patients and the general population in Shanghai by calendar year.

Population	Calendar year	Population at risk	No. of PTB cases	Annual incidence (1/100000)	ASR (1/100000)			Annual incidence in general population (1/100000)	P value*
					Estimate	95% CI			
**All subjects**									
	2010	36 777	17	46.2	67.0	16.9	117.2	29.3	0.058
	2011	72 031	55	76.4	99.9	63.3	136.5	28.9	<0.001
	2012	120 266	63	52.4	73.0	46.1	100.0	28.1	<0.001
	2013	164 145	91	55.4	64.7	42.5	86.9	29.1	<0.001
	2014	199 592	112	56.1	53.6	41.3	65.9	31.0	<0.001
	2015	226 920	101	44.5	50.8	34.4	67.2	29.4	<0.001
**Men**									
	2010	16 530	10	60.5	88.6	8.1	169.2	46.0	0.383
	2011	32 616	43	131.8	156.3	96.6	215.9	44.6	<0.001
	2012	54 753	47	85.8	105.6	66.3	144.9	43.3	<0.001
	2013	74 986	72	96.0	105.4	68.8	142.0	44.7	<0.001
	2014	91 237	86	94.3	87.1	65.7	108.4	47.3	<0.001
	2015	103 798	78	75.2	79.1	54.2	104.0	44.5	<0.001
**Women**									
	2010	20 247	7	34.6	39.5	7.1	71.9	13.2	0.008
	2011	39 415	12	30.5	49.1	10.5	87.8	13.8	0.005
	2012	65 513	16	24.4	44.0	3.5	84.5	13.3	0.014
	2013	89 159	19	21.3	22.8	7.8	37.7	14.0	0.065
	2014	108 356	26	24.0	24.9	11.4	38.4	15.3	0.020
	2015	123 123	23	18.7	25.1	3.2	46.9	14.8	0.259

Abbreviation: T2DM, type 2 diabetes; PTB, pulmonary tuberculosis; ASR: age-standardized rate.

*P values were calculated using a *Z* test.

**Figure 1:**
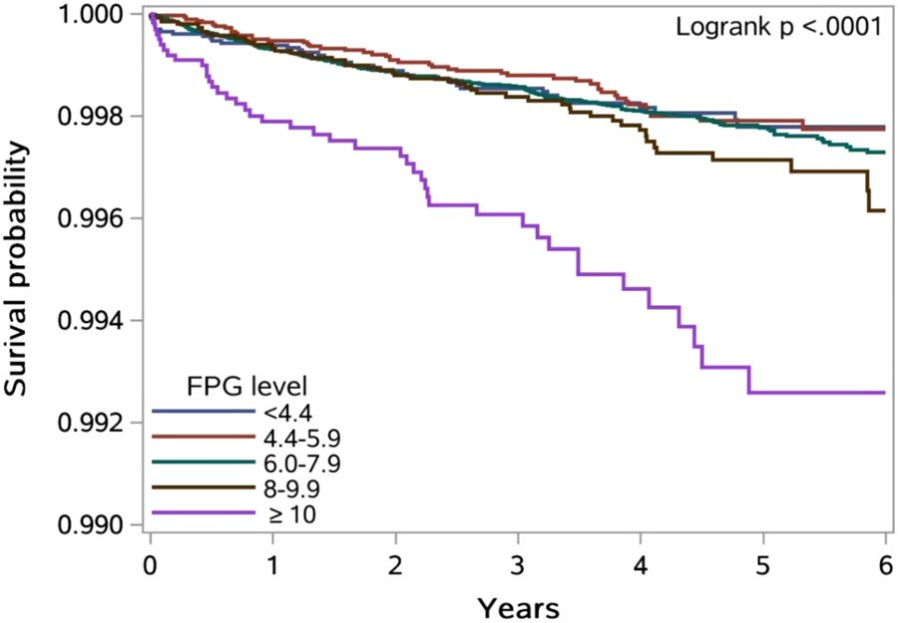
Kaplan-Meier curves of PTB-free survival in T2DM patients by FPG level. Abbreviation: FPG, fasting plasma glucose.

The patients were followed up for 855,782 person-years. As shown in Table 3, the incidence rate of PTB in T2DM patients was 51.3 per 100,000 person-years in all patients, 86.2 per 100,000 person-years in men and 22.1 per 100,000 person-years in women. A significant difference was observed in incidence rate of PTB by age groups, BMI, status of central obesity and FPG level. In particular, incidence of PTB reached 215.2 per 100,000 person-years in T2DM patients with BMI < 18.5 kg/m^2^ (442.0 per 100,000 person-years in men and 77.0 per 100,000 person-years in women), much higher than those in T2DM patients with BMI ≥ 18.5 kg/m^2^. A significantly higher incidence of PTB was also observed in T2DM patients with FPG ≥ 10.0 mmol/L, which was as high as 143.2 per 100,000 person-years in all patients. Moreover, in the Kaplan-Meier plot, there was a significant separation between the subgroup of FPG ≥ 10.0 mmol/L and the other four subgroups (Fig. 1). Interestingly, we did not find a significant difference in the incidence rate of PTB by FPG levels in women.

**Table 3:** Incidence rate of PTB per 100000 person-years among T2DM patients by subgroups.

	All subjects					Men					Women				
	No. of T2DM patients	Person years	No. of PTB cases	Incidence rate (1/100000)	P value*	No. of T2DM patients	Person years	No. of PTB cases	Incidence rate (1/100000)	P value*	No. of T2DM patients	Person years	No. of PTB cases	Incidence rate (1/100000)	P value*
Overall	240 692	855782	439	51.3		110262	389713	336	86.2		130 430	466069	103	22.1	
Age group (years)					<0.001					0.027					0.121
35–44	7779	29150	23	78.9		4841	17937	17	94.8		2938	11213	6	53.5	
45–54	42 250	163970	112	68.3		20 571	78761	90	114.3		21 679	85209	22	25.8	
55–64	89 934	322037	157	48.8		40 610	144765	117	80.8		49 324	177272	40	22.6	
65–74	60 309	207232	93	44.9		27 700	94633	74	78.2		32 609	112599	19	16.9	
75–	40 420	133393	54	40.5		16 540	53617	38	70.9		23 880	79776	16	20.1	
BMI (kg/m^2^) ^a^					<0.001					<0.001					<0.001
<18.5	3491	12548	27	215.2		2140	4752	21	442.0		1351	7796	6	77.0	
18.5–24.9	139 819	496125	321	64.7		74 468	230843	252	109.2		65 351	265281	69	26.0	
≥25.0	91 108	329073	85	25.8		50 530	145608	58	39.8		40 578	183465	27	14.7	
Central obesity ^b^					<0.001					<0.001					0.010
Yes	100 940	367961	132	35.9		42 155	154020	97	63.0		58 785	213941	35	16.4	
No	128 844	458011	294	64.2		62 730	220968	228	103.2		66 114	237042	66	27.8	
High blood pressure ^c^					0.573					0.841					0.106
Yes	45 833	159765	78	48.8		22 311	77590	66	85.1		23 522	82175	12	14.6	
No	191 071	683430	357	52.2		86 145	306171	267	87.2		104 926	377259	90	23.9	
FPG (mmol/L) ^d^					<0.001					<0.001					0.245
<4.4	554	2017	1	49.6		271	1000	1	100.0		283	1018	0	0	
4.4–5.9	29 235	113187	47	41.5		12 652	48308	33	68.3		16 583	64879	14	21.6	
6.0–7.9	152 066	541679	260	48.0		68 481	243693	200	82.1		83 585	297985	60	20.1	
8–9.9	26 188	87119	51	58.5		12 744	42131	39	92.6		13 444	44988	12	26.7	
≥10	9956	30031	43	143.2		5307	15753	36	228.5		4649	14277	7	49.0	
Interval between diagnosis and registry (years) †					0.378					0.569					0.353
<0.5	92 954	231161	115	49.8		42 660	104721	88	84.0		50 294	126440	27	21.4	
0.5–0.9	34 267	119121	65	54.6		14 973	51336	51	99.4		19 294	67786	14	20.7	
1–1.4	27 901	100759	50	49.6		12 817	45836	41	89.5		15 084	54922	9	16.4	
1.5–1.9	19 589	85666	54	63.0		8876	38509	38	98.7		10 713	47158	16	33.9	
≥2	65 981	319074	155	48.6		30 936	149311	118	79.0		35 045	169763	37	21.8	

Abbreviation: T2DM, type 2 diabetes; PTB, pulmonary tuberculosis; BMI, body mass index; FPG, fasting plasma glucose.

*P values were calculated using a Log-rank test.

^†^Referring to interval between date of diagnosis of T2DM and date of registration in Shanghai Standardized Diabetes Management System.

^a^6274 missing values excluded from analysis; ^b^10908 missing values excluded from analysis; ^c^3788 missing values excluded from analysis; ^d^22693 missing values excluded from analysis.

**Figure 2:**
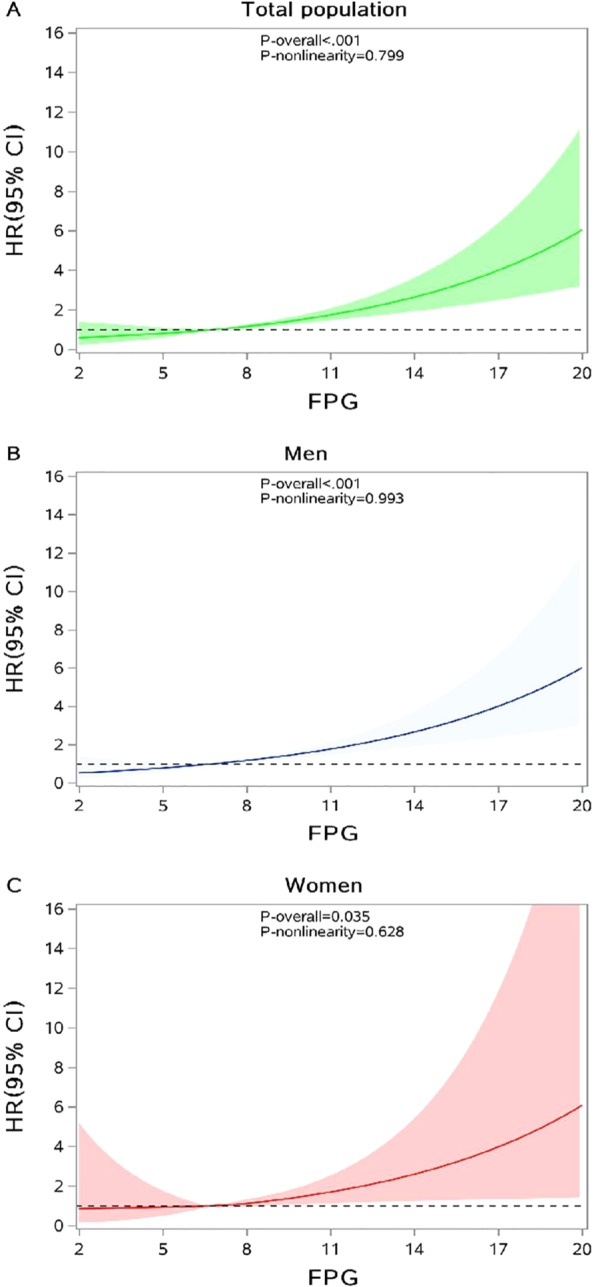
Association between FPG and risk of PTB among all T2DM patients(**A**), men (**B**) and women (**C**). The reference of FPG for these plots (with HR fixed as 1.0) was 6.8 mmol/L. HR was adjusted for age, sex, BMI. Abbreviations: FPG, fasting plasma glucose; HR, hazard ratio; CI: confidence interval.

**Figure 3:**
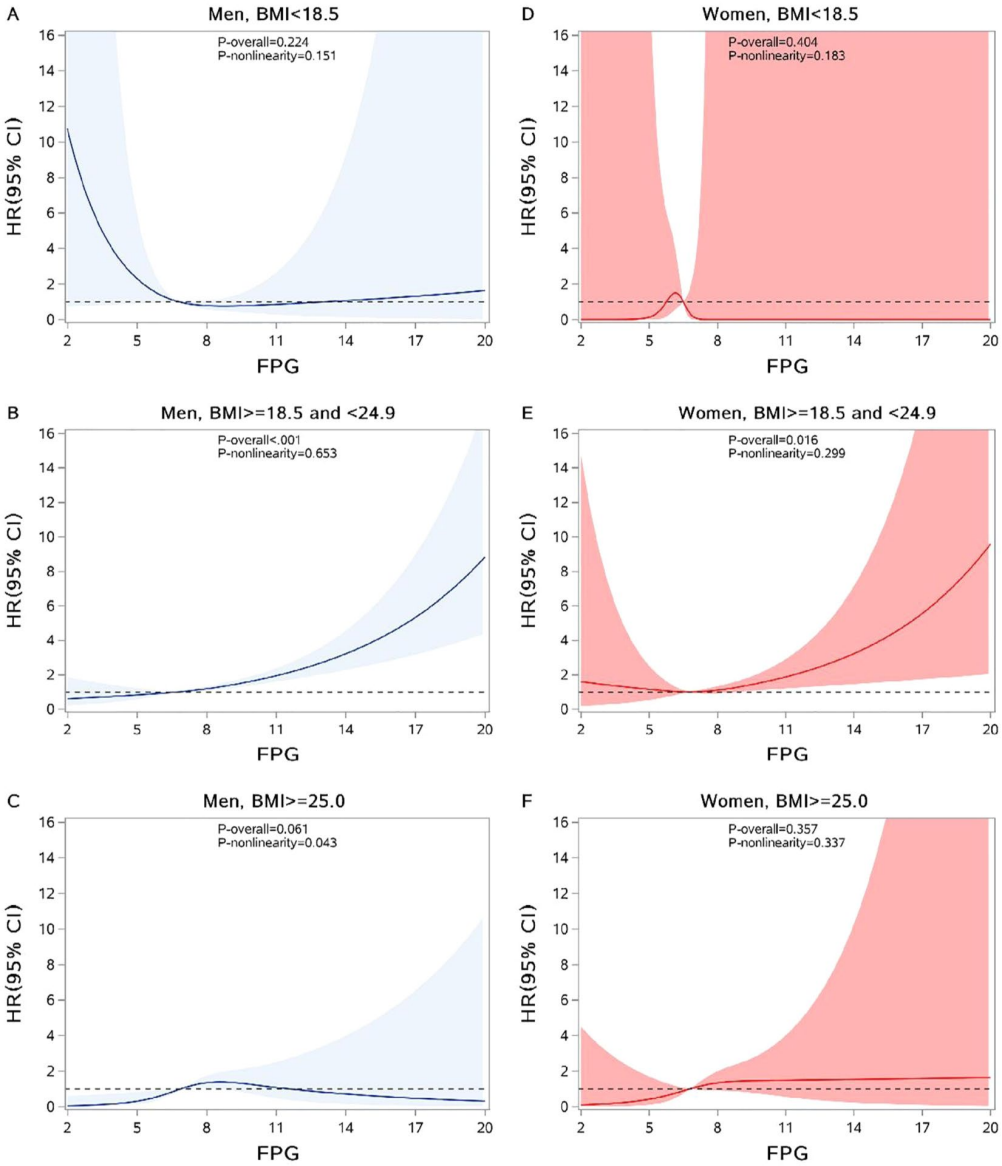
Association between FPG and risk of PTB incidence among men with BMI < 18.5 kg/m^2^ (**A**), men with BMI between 18.5 and 24.9 kg/m^2^ (**B**), men with BMI ≥ 25.0 kg/m^2^ (**C**), women with BMI < 18.5 kg/m^2^ (**D**), women with BMI between 18.5 and 24.9 kg/m^2^ (**E**) and women with BMI ≥ 25.0 kg/m^2^ (**F**). The reference of FPG for these plots (with HR fixed as 1.0) was 6.8 mmol/L. HR was adjusted for age. Abbreviations: FPG, fasting plasma glucose; BMI, body mass index; HR, hazard ratio; CI, confidence interval.

Further analysis was conducted to evaluate the association of FPG level with the risk of PTB in T2DM patients. After adjustment for sex, age and BMI, a significantly linear relationship between FPG and the risk of PTB incidence was observed in all subjects (Table 4; Fig. 2). When FPG was used as a categorical variable, the risk of PTB incidence in patients with high FPG level was 2.70 (95% CI: 1.95, 3.73) times higher than those with relatively normal FPG level (Table 4). The similar association pattern still existed in men and women separately. Since we found the interaction between BMI level and FPG level (P = 0.029), a stratified analysis by BMI level was performed. The significantly linear association of FPG and risk of PTB incidence only persisted in normal-weight patients both in men and women (Fig. 3). For underweight and overweight or obese patients, either significantly non-linear relationship or linear relationship was not observed.

**Table 4:** Multivariable-adjusted hazard ratios and 95% confidence interval of PTB according to FPG.

Categories of FPG	HR (95% CI)		
	All subjects*	Men^†^	Women^†^
FPG as a continuous variable	1.14 (1.09,1.19)	1.15 (1.09, 1.20)	1.12 (1.01, 1.24)
**FPG level**			
<4.4	0.92 (0.64,1.31)	0.87 (0.57,1.31)	1.11 (0.55, 2.30)
4.4–5.9	0.89 (0.65, 1.21)	0.84 (0.58, 1.21)	1.04 (0.58, 1.87)
6.0–7.9	1.00	1.00	1.00
8–9.9	1.21 (0.90, 1.64)	1.17 (0.83, 1.65)	1.37 (0.74, 2.55)
≥10	2.70 (1.95, 3.73)	2.74 (1.92, 3.92)	2.46 (1.12, 5.38)

Abbreviation: FPG, fasting plasma glucose; PTB, pulmonary tuberculosis; HR, hazard ratio; CI, confidence interval.

*Adjusted for age, sex and BMI.

^†^Adjusted for age and BMI.

## Discussion

Although the association of TB and T2DM has been verified in several studies^5,9,16,25^, we noted that population-based cohort studies focusing on the risk of TB incidence among T2DM patients in Shanghai, China, were limited, where the burden of diabetes is soaring year by year. In our study, we found that the annual incidence of PTB remained higher than that in general population from 2010 to 2015, and the overall incidence rate was 51.3 per 100,000 person-years. There was a substantially higher incidence rate among patients with FPG ≥ 10 mmol/L (143.2 per 100,000 person-years). Especially in diabetes patients of normal weight, the risk of PTB incidence increased with increasing FPG.

The incidence of PTB was approximately 2–3 times higher in T2DM patients aged 35 or above than in the general population in Shanghai, China, from 2010 to 2015. Our results are consistent with a prospective study conducted in Dhaka, which reported that the incidence rate of confirmed PTB cases among DM patients was double than that observed in the general population^26^. Another prospective study, conducted in Yunnan province, China, demonstrated that the rate of TB incidence was almost 3 times higher than that found in the general population^12^. A retrospective cohort study in the English population also reported a similar result and found DM was associated with a more than twofold increased risk of PTB^27^. Additionally, compared to previous studies, overall incidence rate of TB in T2DM patients in Shanghai was much lower than other studies conducted in China but was higher than some studies in western countries. In a cohort study conducted in Taiwan, the cumulative incidence of TB was 1.92 cases per 1000 person-years for females and 3.25 cases per 1000 person-years for males^28^. A large UK population-based cohort study reported that the incidence of TB among patients with diabetes was 16.2 per 100,000 person-years^16^.

Our study found the incidence rate of PTB among T2DM patients was influenced by FPG level. The highest overall rate was 143.2 per 100,000 person-years in the subgroup of FPG ≥ 10.0 mmol/L. Furthermore, the risk of PTB incidence increased as FPG increased, presenting a linear relationship, which was consistent with a cohort study conducted in Taiwan^18^. However, not all the reported results were in line with ours. The aforementioned UK study reported that there was no statistically significant difference of incidence rate of TB among three subgroups of hemoglobin A1c^16^. Leegaard *et al*. conducted a population-based case-control study in Northern Denmark and found no statistically significant evidence for any association between TB and dysglycemia^17^. To explain the negative results, these two studies both mentioned that one of the limitations of their studies was inadequate adjustments for important individual level confounders from lifestyle and demographic risk factors such as BMI, which had a strong and consistent long-linear relationship with TB incidence^23^. Besides, in the UK cohort study, most of the patients had reasonably well-controlled diabetes, which may cause underestimation and could reflect that good glycaemic control could decrease the incidence risk of tuberculosis.

As mentioned above, a number of previous studies have verified BMI and TB incidence presented an inverse relationship^23^. Similarly, in our study, the incidence rates of PTB were 215.2, 64.7 and 25.8 per 100,000 person-years in underweight patients, normal-weight patients and overweight/obese patients, respectively, presenting a significantly decreasing tendency. When grouped by sex, this trend still existed. These results are consistent with two population-based cohort studies conducted in Taiwan^29^.

Therefore, on the basis of the aforementioned inverse relationship and the assumption that overweight and obesity are major drivers of T2DM, it is necessary to take BMI into consideration when it comes to the association between TB incidence risk and FPG. We found that a significantly linear relationship between risk of PTB incidence and FPG was only observed in normal-weight patients regardless of sex. In overweight patients, the FPG level did not have an impact on the incidence of TB, which means that the protective effect of high BMI may be stronger than the increase in risk due to diabetes. Lin *et al*. found that the overall association between BMI and TB risk was dominated by the direct protective effect of BMI that was not mediated through diabetes^29^. Nevertheless, the conclusion was not consistent in all studies. Kubiak *et al*. found that the burden of active TB associated with diabetes was similar for normal and overweight or obese adults in the Indian population^30^. However, they also stressed the finding may not be generalizable beyond the Indian population, who have a higher likelihood of developing diabetes at every BMI level. In our study, the significant association between FPG level and PTB risk was not observed among underweight T2DM patients either. Based on abundant epidemiological evidence that has ascertained the harmful effect of poor nutritional status (or low BMI) on TB incidence^31,32^, we may assume that such an effect also dominated in the progress of TB incidence in T2DM patients instead of FPG level, which is consistent with a prospective cohort study in Singapore reporting that underweight and T2DM were independent determinants for active TB^33^.

The major strengths of our study are as follows. To the best of our knowledge, it is the most representative and large-sample study in China to investigate the incidence rate of PTB by subgroups in T2DM patients and the association between PTB incidence and FPG grouped by BMI level. Furthermore, it is the first study to analyse the detailed incidence rate of PTB and association between PTB incidence and specific FPG level in diabetic patients in Shanghai. Moreover, making full use of information systems, including a diabetes management system and a TB surveillance and mandatory reporting system, ensured the authenticity and validity of the results.

Although we believe that our results shed light on the incidence of PTB in T2DM patients aged 35 or above in Shanghai, they still have several limitations. Since it is a retrospective study, reliable data of potential risk factors related to TB incidence, such as medication use, smoking history, alcohol consumption and physical activity^34,35^, were not available from the current system. Second, not all the T2DM patients were registered in the management system, which covered about half of all T2DM patients^10^. Selection bias existed because the registered DM cases were mainly identified through screenings and outpatient visits to community health centres. Elderly people tend to participate in screenings and visit community health centres, while young and middle-aged adults prefer the academic hospitals. It is possible that DM patients who were not registered were at greater risk of poor glycaemic status as well as greater risk of TB. Third, the information on glucose was based on a single FPG test at baseline, and whether it could reflect the individuals’ real glycaemic status when they were diagnosed with TB may be unclear. Once more accurate and reasonable data are available through future studies, we will conduct re-analysis of this issue. Fourth, the number of underweight T2DM patients was relatively rare in our study; thus, the curvilinear shape of the dose-response relationship in underweight patients was a little strange, though it was not significant, especially in women. Finally, T2DM patients enrolled near the end of the study would not have time to develop TB, which may underestimate the incidence of TB slightly. However, since it was a sustainable cohort and we will follow up those cases, complete analysis will be possible in future studies.

In summary, our study revealed that the increasing prevalence of diabetes has had a significant influence on the incidence rate of TB, which is higher in T2DM patients than in the general population. Our results also imply that the next step of screening for TB in diabetics should focus on determining and prioritizing the high-risk T2DM patients in order to improve cost-effectiveness. For normal-weight diabetics, those with very poor glycaemic control would be a targeted screening population, while it may not be cost-effective to provide extra screening for those with good glycaemic control. For underweight diabetics, improvement of nutritional status would be more vital instead of strict glycaemic control. For overweight or obese diabetics, finding the balance point of glycaemic control and appropriate BMI would be the key to preventing TB and complications of diabetes at the same time.
